# *Arsenophonus* Interacts with *Buchnera* to Improve Growth Performance of Aphids under Amino Acid Stress

**DOI:** 10.1128/spectrum.01792-23

**Published:** 2023-05-24

**Authors:** Pan-Pan Tian, Yu-Lin Zhang, Jing-Ling Huang, Wang-Yan Li, Xiang-Dong Liu

**Affiliations:** a Department of Entomology, Nanjing Agricultural University, Nanjing, China; Connecticut Agricultural Experiment Station

**Keywords:** *Arsenophonus*, *Aphis gossypii*, *Buchnera*, growth performance, amino acid synthase, gene expression, symbiont abundance

## Abstract

Amino acids play a crucial role in the growth and development of insects. Aphids cannot ingest enough amino acids in plant phloem to meet their requirements, and therefore, they are mainly dependent on the obligate symbiont Buchnera aphidicola to synthesize essential amino acids. Besides *Buchnera*, aphids may harbor another facultative symbiont, *Arsenophonus*, which alters the requirement of the cotton-melon aphid Aphis gossypii for amino acid. However, it is unclear how *Arsenophonus* regulates the requirement. Here, we found that *Arsenophonus* ameliorated growth performance of *A. gossypii* on an amino acid-deficient diet. A deficiency in lysine (Lys) or methionine (Met) led to changes in the abundance of *Arsenophonus*. *Arsenophonus* suppressed the abundance of *Buchnera* when aphids were fed a normal amino acid diet, but this suppression was eliminated or reversed when aphids were on a Lys- or Met-deficient diet. The relative abundance of *Arsenophonus* was positively correlated with that of *Buchnera*, but neither of them was correlated with the body weight of aphids. The relative expression levels of Lys and Met synthase genes of *Buchnera* were affected by the interaction between *Arsenophonus* infections and *Buchnera* abundance, especially in aphids reared on a Lys- or Met-deficient diet. *Arsenophonus* coexisted with *Buchnera* in bacteriocytes, which strengthens the interaction.

**IMPORTANCE** The obligate symbiont *Buchnera* can synthesize amino acids for aphids. In this study, we found that a facultative symbiont, *Arsenophonus*, can help improve aphids’ growth performance under amino acid deficiency stress by changing the relative abundance of *Buchnera* and the expression levels of amino acid synthase genes. This study highlights the interaction between *Arsenophonus* and *Buchnera* to ameliorate aphid growth under amino acid stress.

## INTRODUCTION

Insects often harbor several species of symbionts which play important roles in fitness and behavior of host populations (1–5). Aphids are small sap-sucking insects which depend on symbionts to meet the challenge of low quantity and considerable imbalance of amino acids in food. The obligate symbiont Buchnera aphidicola provides aphids with amino acids and vitamin B that are usually scarce in the phloem of plants (6–8). Therefore, this symbiont has become indispensable to aphids, having established symbiosis 160 to 280 million years ago (9). Besides the obligatory symbiont, aphids may harbor other facultative endosymbionts, “*Candidatus* Regiella insecticola,” “*Candidatus* Hamiltonella defensa,” Serratia symbiotica, etc., which are not necessary but affect fitness of host populations with regard to characteristics such as heat tolerance (10), resistance to enemies (11), suppression of plant defense (12, 13), and nutritional benefits (14). The coinfection of aphids by *Buchnera* with a facultative symbiont is very common (15–20).

The interaction between an obligate and a facultative symbiont may occur due to coexistence in a host. It has been found that the facultative endosymbiont Serratia symbiotica could partially compensate for the role of *Buchnera* to maintain the survival and reproduction of the pea aphid Acyrthosiphon pisum when *Buchnera* was removed (21), and it might have performed the transition from a facultative endosymbiont to an obligate one in the aphid Cinara cedri (22). The facultative endosymbiont “*Ca.* Hamiltonella defensa” might improve Sitobion miscanthi fitness via stimulating the proliferation of *Buchnera* (23).

All cotton-melon aphid Aphis gossypii individuals harbor the obligate symbiont *Buchnera*, and 30 to 75% of them also harbor the facultative symbiont *Arsenophonus* (14, 16, 24–27). *Arsenophonus* is associated with growth performance of aphids. *Arsenophonus* infections overall benefit the soybean aphid Aphis glycines in population growth, but this benefit is dependent on aphid clone, plant, and time (28). This symbiont is also associated with the host range of the cowpea aphid, Aphis craccivora, and aphids infected with *Arsenophonus* can use black locust but cannot use alfalfa (29). *Arsenophonus* infections increase the survival and reproduction of the cotton-melon aphid and alter requirements of aphids for amino acids in their diet (14, 16, 30). On the other hand, the abundance of the obligate symbiont *Buchnera* may be affected by the *Arsenophonus*. It has been found that the *Buchnera* population was suppressed by the presence of the facultative endosymbiont *Rickettsia* or *S. symbiotica* in the pea aphid (21, 31). The abundance of *Buchnera* was reduced at a rate similar to that of *Arsenophonus* in the soybean aphid *Aphis glycines* when aphids were raised on a resistant host plant and exposed to a pesticide (32). In the cotton-melon aphid, coinfection with *Arsenophonus* and “*Candidatus* Hamiltonella” did not affect the relative abundance of *Buchnera* (16), but the *Buchnera* population sizes and *Arsenophonus* infection rates in aphids on different host plants were significantly different (14, 33). Therefore, the relationship between *Buchnera* and *Arsenophonus* still needs study.

Aphis gossypii populations commonly exhibit host specialization on different host plants, and cucurbit- and cotton-specialized biotypes exist worldwide (34–36). Contents of free amino acids in cotton leaves are lower than in cucumber leaves (14, 37), indicating that the cotton- and cucurbit-specialized aphids undergo different nutrient stresses. Moreover, the cotton- and cucurbit-specialized aphids are also infected with *Arsenophonus* at different rates, and the infection rate in aphids on cotton is significantly higher than that in aphids on cucurbits (14, 27). *Arsenophonus* infections alter the requirements of cotton-melon aphids for some specific amino acids (14), which may contribute to the development of host specialization in aphid populations, but how *Arsenophonus* affects this requirement of aphids is still ambiguous. The effect of a facultative endosymbiont on aphids may be dependent on aphid genotype (18, 28). Therefore, as we focused on the amino acid provider *Buchnera* (6–8), we hypothesized that *Arsenophonus* ameliorates aphid performance under amino acid stress via interaction with *Buchnera* and that this role might be dependent on aphid genetic background. In this study, we measured amino acid titers in three genotypes of *A. gossypii* infected with and cured of *Arsenophonus*. Then, we examined growth performance of these three genotypes infected with and cured of *Arsenophonus* on diets with deficient and excessive amino acids and detected the effects of *Arsenophonus* infections on the relative abundance and gene expression levels of *Buchnera* related to amino acid synthase. Finally, we detected the location of *Arsenophonus* and *Buchnera* in aphids. The results showed that *Arsenophonus* infections affected the titer of amino acid in aphids and growth performance of aphids under amino acid stress and that these effects were mediated by the interaction between *Arsenophonus* and *Buchnera* with regard to endosymbiont abundance and expression level of the amino acid synthase gene.

## RESULTS

### Effect of *Arsenophonus* infection on amino acid titers in aphids.

**FIG 1:**
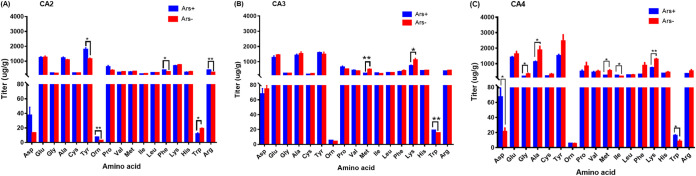
Titers of 17 amino acids in the *Arsenophonus*-infected (Ars^+^) and cured (Ars^−^) lines of aphid genotypes CA2 (A), CA3 (B), and CA4 (C). Differences between Ars^+^ and Ars^−^ lines were analyzed by Student’s *t* test. *, *P* = 0.05; **, *P* = 0.01.

The titer of amino acid in aphids was significantly dependent on the interaction between *Arsenophonus* infection and aphid genotype (Roy’s largest root = 532.552, *P* = 0.011) (see Table S2 in the supplemental material), and titers of 10 (Asp, Gly, Ala, Tyr, Orn, Met, Phe, Lys, Trp, and Arg) out of 17 amino acids were significantly affected by the interaction (Fig. 1; Table S3). The *Arsenophonus* infection decreased titers of Met and Lys in aphids, especially in the CA3 (Met: *t *= 5.905, *df *= 4, *P* = 0.004; Lys: *t *= 2.928, *df *= 4, *P* = 0.043) (Fig. 1B) and CA4 (Met: *t *= 4.719, *df *= 4, *P* = 0.036; Lys: *t *= 6.757, *df *= 4, *P* = 0.003) (Fig. 1C) genotypes.

### Effect of *Arsenophonus* infection on aphid growth performance.

**FIG 2:**
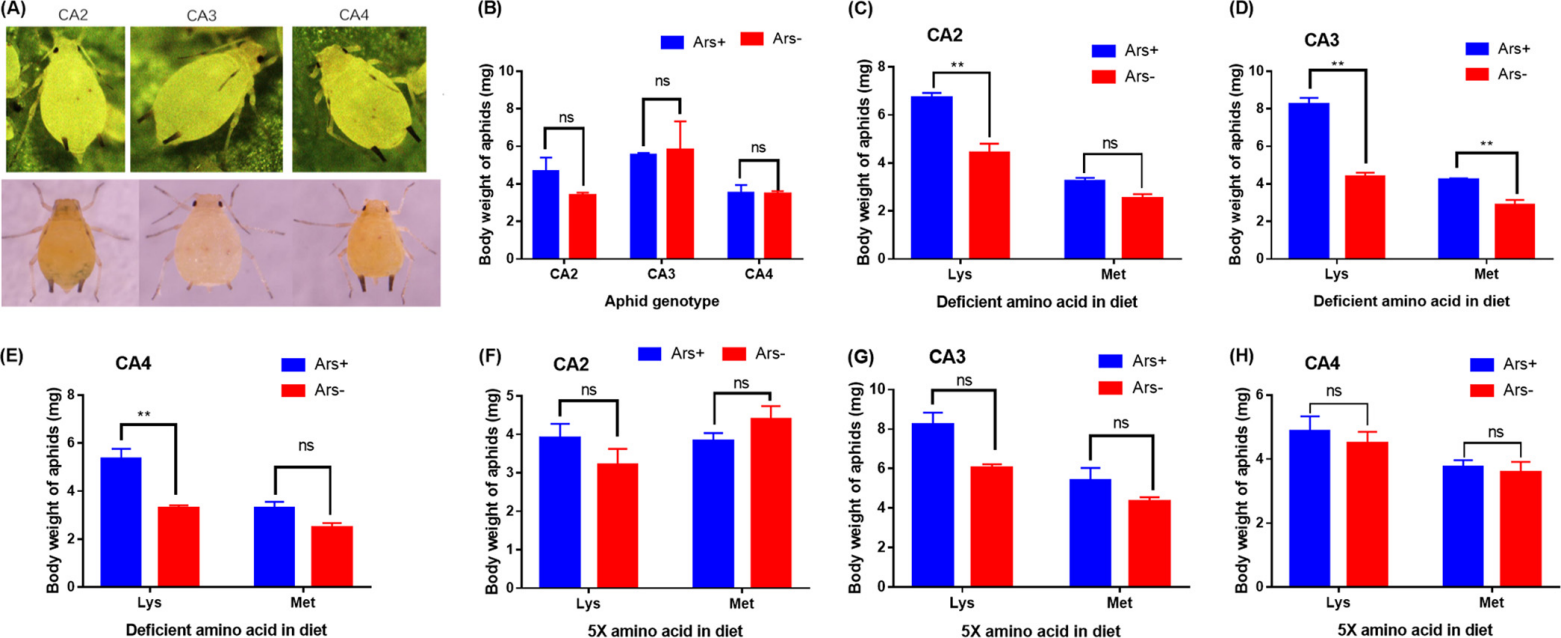
CA2, CA3, and CA4 aphids (A) and body weights of 100 3-day-old *Arsenophonus*-infected and cured CA2, CA3, and CA4 aphids reared on the 1× amino acid diet (B), Lys or Met omission diet (C to E), and 5× Lys or Met diet (F to H). Differences in body weights between Ars^+^ and Ars^−^ lines were analyzed by Student’s *t* test followed by the Bonferroni correction. *, *P* = 0.05; **, *P* = 0.01; ns, no significant difference (*P* = 0.05).

The effect of *Arsenophonus* infection on aphid growth performance was dependent on which amino acids were present in the diet. *Arsenophonus* infections did not affect body weights of the CA2 (*t *= 1.422, *df *= 5, *P* = 0.214), CA3 (*t *= 0.174, *df *= 4, *P* = 0.871), and CA4 (*t *= 0.055, *df *= 6, *P* = 0.958) aphids when these aphids (Fig. 2A) were reared on the artificial diet with a normal concentration (1×) of amino acids (Fig. 2B). However, when CA2 (*t *= 5.426, *df *= 5, *P* = 0.018), CA3 (*t *= 9.854, *df *= 5, *P* = 0.0012), and CA4 (*t *= 5.289, *df *= 5, *P* = 0.0192) aphids were reared on a diet with only Lys omitted, body weights of *Arsenophonus*-infected lines were significantly higher than those of their *Arsenophonus*-cured counterparts (Fig. 2C to E). On the diet with only Met omitted, the body weights of CA3 aphids infected with *Arsenophonus* were also significantly higher than those of the *Arsenophonus*-cured line (*t *= 4.251, *df *= 6, *P* = 0.030) (Fig. 2D), whereas the body weights of *Arsenophonus*-infected CA2 (*t *= 2.996, *df *= 6, *P* = 0.134) (Fig. 2C) and CA4 (*t *= 2.313, *df *= 6, *P* = 0.36) (Fig. 2E) aphids were similar to those in the *Arsenophonus*-cured lines. On the 5× Lys or Met diet, *Arsenophonus* infections did not affect the body weights of aphids (Fig. 2F to H).

### Response of *Arsenophonus* in aphids to amino acid stress in the diet.

**FIG 3:**
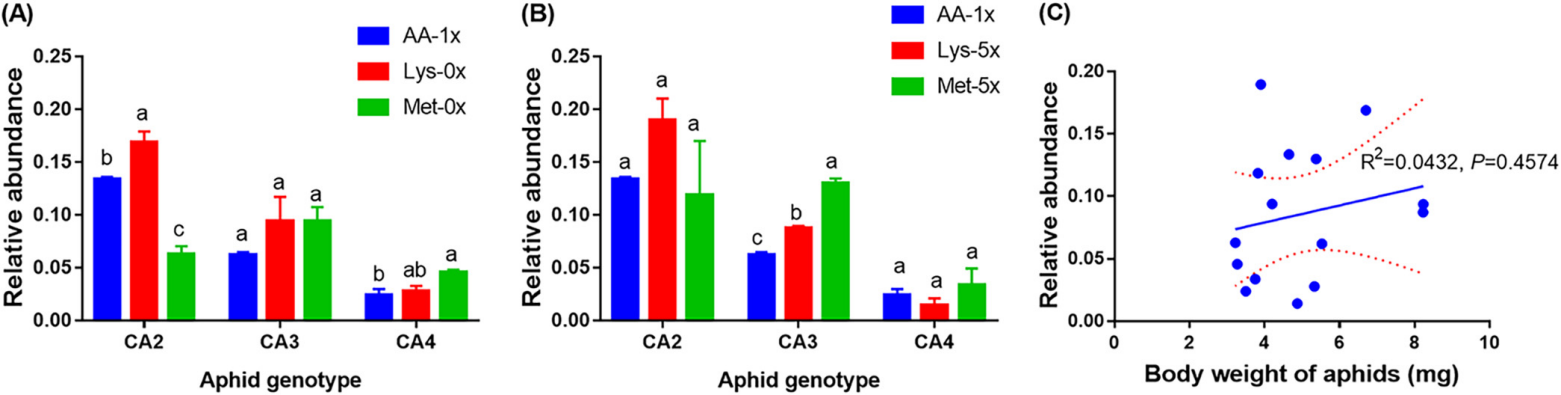
Effects of Lys- or Met-deficient (0×) (A) and excess (5×) (B) diets on the relative abundance of *Arsenophonus* in 3-day-old aphids and the relationship between relative abundance of *Arsenophonus* and body weight of aphids (C). The solid line is the linear regression line, and the dashed lines show the 95% confidence intervals. Different lowercase letters indicate significant differences in the relative abundance of *Arsenophonus* among the same genotype of aphids reared on different diets (*P* = 0.05), analyzed by ANOVA followed by the *post hoc* Tukey’s test.

Amino acid deficiency (0×) in the diet significantly affected the relative abundance of *Arsenophonus* in aphids (*F*_2,18_ = 6.764, *P* = 0.006) (Fig. 3A), but amino acid excess in the diet did not (*F*_2,18_ = 1.321, *P* = 0.292) (Fig. 3B). The relative abundance of *Arsenophonus* increased in aphids reared on the Lys-deficient diet, especially in the CA2 line (Fig. 3A). The aphid genotype also significantly affected the relative abundance of *Arsenophonus*, on either the amino acid-deficient diet (*F*_2,18_ = 56.038, *P < *0.001) (Fig. 3A) or the amino acid excess diet (*F*_2,18_ = 29.884, *P < *0.001) (Fig. 3B). The amino acid deficiency affected the relative abundance of *Arsenophonus* in the CA2 (*F*_2,6_ = 51.632, *P < *0.001) and CA4 (*F*_2,6_ = 6.251, *P* = 0.034) lines but did not affect the abundance of the CA3 line (*F*_2,6_ = 1.383, *P* = 0.321). In contrast, the amino excess did not affect the relative abundance of *Arsenophonus* in CA2 (*F*_2,6_ = 1.356, *P* = 0.327) and CA4 (*F*_2,6_ = 0.877, *P* = 0.463) lines but affected the CA3 line (*F*_2,6_ = 100.637, *P < *0.001). When all data collected from *Arsenophonus*-infected CA2, CA3, and CA4 aphids reared on all three types of diet (0×, 1×, and 5× Lys and Met) were pooled, the body weights were not significantly correlated with the relative abundance of *Arsenophonus* (*r *= 0.2078, *df *= 13, *P* = 0.4574) (Fig. 3C), suggesting that the growth performance of aphids harboring *Arsenophonus* is not wholly mediated by the abundance of this endosymbiont.

### Effects of *Arsenophonus* infection on *Buchnera* abundance.

**FIG 4:**
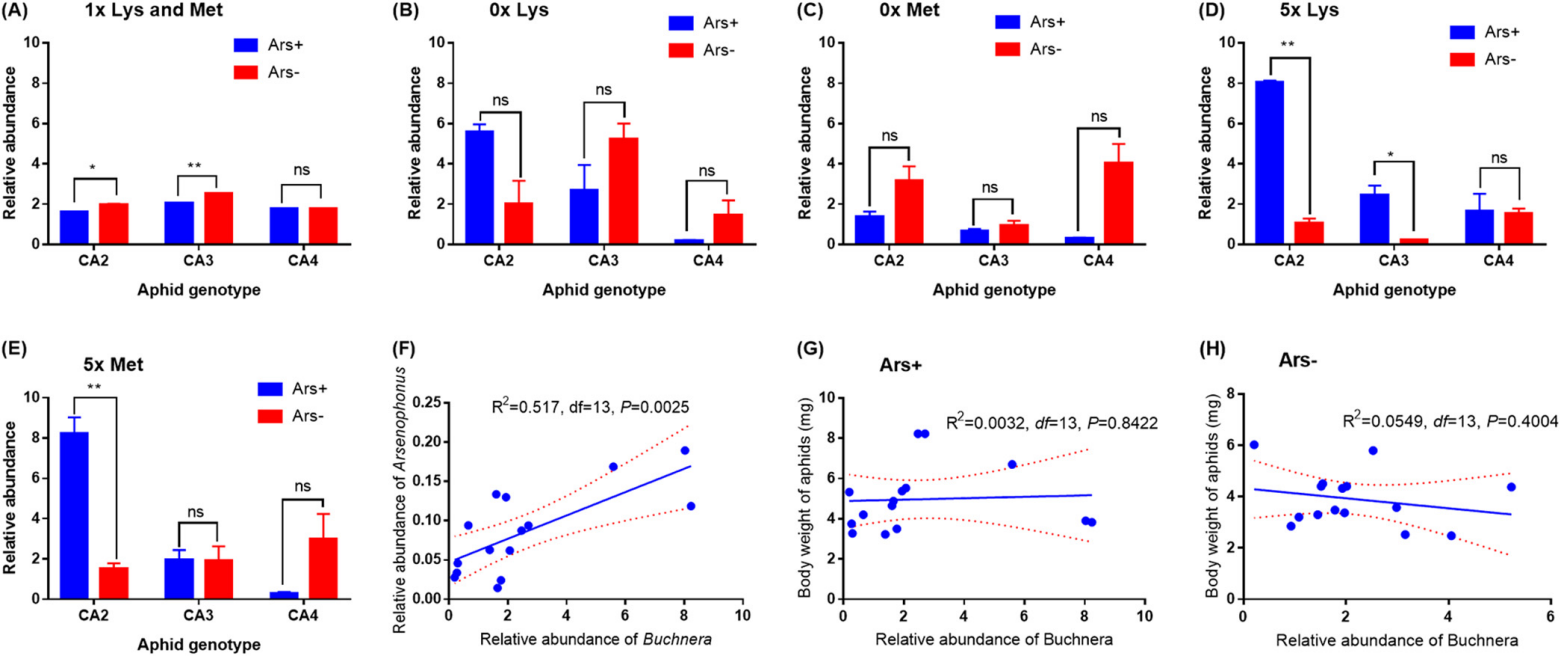
Relative abundance of *Buchnera* in aphids reared on the 1× (A), 0× (B and C), and 5× (D and E) Lys and Met diets and the correlation between relative abundance of *Buchnera* and *Arsenophonus* (F) and between relative abundance of *Buchnera* and body weight of aphids infected with (G) and cured of (H) *Arsenophonus*. The solid line is the linear regression line, and the dashed lines show the 95% confidence intervals. Differences in the relative abundance of *Buchnera* in aphids between Ars^+^ and Ars^−^ lines were analyzed by Student’s *t* test followed by the Bonferroni correction. *, *P* = 0.05; **, *P* = 0.01; ns, no significant difference (*P* = 0.05).

*Arsenophonus* infection (*F*_1,12_ = 165.45, *P < *0.001) and aphid genotype (*F*_2,12_ = 255.558, *P < *0.001) affected the relative abundance of *Buchnera* (Fig. 4A). On the 1× Lys and Met diet, the relative abundance of *Buchnera* in the *Arsenophonus*-infected CA2 (*t *= 6.048, *df *= 4, *P* = 0.0113) and CA3 (*t *= 21.189, *df *= 4, *P < *0.001) aphids was significantly lower than that in the *Arsenophonus*-cured lines (Fig. 4A), suggesting the suppression of *Buchnera* by *Arsenophonus*. However, when those aphids were reared on the Lys- or Met-deficient diet, the relative abundance of *Buchnera* in the *Arsenophonus*-infected CA2 (Lys-deficient diet: *t *= 2.948, *df *= 4, *P* = 0.126; Met-deficient diet: *t *= 2.315, *df *= 4, *P* = 0.246) and CA3 (Lys-deficient diet: *t *= 1.715, *df *= 4, *P* = 0.483; Met-deficient diet: *t *= 0.966, *df *= 4, *P* = 1.00) aphids became similar to that in the *Arsenophonus*-cured aphids (Fig. 4B and C), indicating the removal of the suppression. On the 5× Lys diet, the relative abundance of *Buchnera* in the *Arsenophonus*-infected CA2 (*t *= 29.493, *df *= 4, *P < *0.001) and CA3 (*t *= 4.869, *df *= 4, *P* = 0.0247) lines was significantly higher than that in the *Arsenophonus*-cured lines (Fig. 4D), as for CA2 on the 5× Met diet (*t *= 8.019, *df *= 4, *P* = 0.003) (Fig. 4E). The relative abundance of *Buchnera* in CA4 aphids was not affected by the *Arsenophonus* infection (Fig. 4). Upon pooling all data collected from CA2, CA3, and CA4 aphids reared on the 0×, 1×, and 5× Lys and Met diets, we found that there was significant positive correlation between the relative abundance of *Buchnera* and *Arsenophonus* in aphids (*r *= 0.719, *df *= 13, *P* = 0.0025) (Fig. 4F), but there was no significant correlation between the relative abundance of *Buchnera* and body weight of the *Arsenophonus*-infected aphids (*r *= 0.056, *df *= 13, *P* = 0.8422) (Fig. 4G) and *Arsenophonus*-cured aphids (*r* = −0.234, *df *= 13, *P* = 0.4004) (Fig. 4H), suggesting that the growth performance of aphids under amino acid stress is not wholly determined by the abundance of *Buchnera*.

### *Arsenophonus* affects the expression level of the amino acid synthase gene of *Buchnera*.

**FIG 5:**
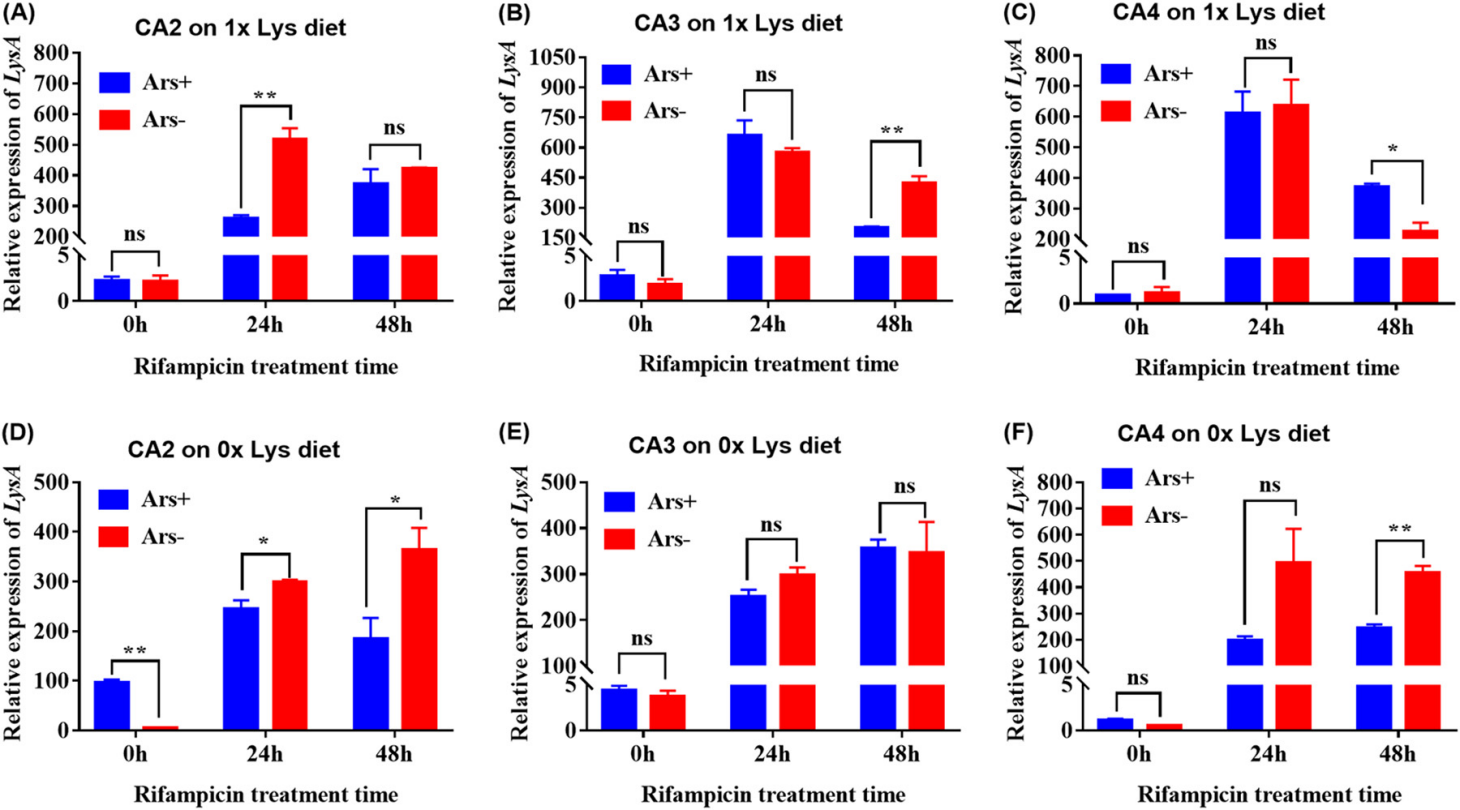
Effect of *Arsenophonus* infection on the expression level of the amino acid synthase gene *lysA* of *Buchnera* in aphids reared on the 1× (A to C) and 0× (D to F) Lys diets when their *Buchnera* levels were normal (no rifampicin treatment [0 h]) and reduced by using rifampicin for 24 h and 48 h. Differences in the relative expression level of the *Buchnera* gene between the Ars^+^ and Ars^−^ lines were analyzed by Student’s *t* test. *, *P* = 0.05; **, *P* = 0.01; ns, no significant difference (*P* = 0.05).

The effect of *Arsenophonus* on the expression level of *lysA*, a Lys synthase gene, in *Buchnera* was dependent on *Buchnera* abundance and amino acid in diet (Fig. 5). When *Buchnera* was normal without antibiotic treatment (0 h), *Arsenophonus* infections did not affect the relative expression levels of *lysA* in the CA2 (*t *= 0.129, *df *= 4, *P* = 0.904), CA3 (*t *= 0.934, *df *= 4, *P* = 0.403), and CA4 (*t *= 0.471, *df *= 4, *P* = 0.661) aphids on the 1× Lys diet (Fig. 5A to C), but on the 0× Lys diet, *Arsenophonus* infections increased the relative expression level of *lysA*, and notably, the increase in the CA2 line was significant (*t *= 13.663, *df *= 4, *P* = 0.00017) (Fig. 5D). In contrast, when *Buchnera* was partially eliminated by antibiotic treatment for 24 h (*t *= 2.992, *df *= 4, *P* = 0.040) and 48 h (*t *= 2.918, *df *= 4, *P* = 0.043), *Arsenophonus* infections significantly decreased the relative expression level of *lysA* in CA2 aphids reared on the 0× Lys diet (Fig. 5D). The interaction of *Arsenophonus* with *Buchnera* based on time of antibiotic treatment significantly affected the relative expression level of *lysA* in CA2 aphids on the Lys-deficient diet (*F*_2,12_ = 13.37, *P* = 0.001) (Fig. 5D). *Buchnera* reduction significantly increased the relative expression levels of *lysA* in the CA2, CA3, and CA4 lines, regardless of whether aphids were on the 0× (CA2: *F*_2,12_ = 48.252, *P < *0.001; CA3: *F*_2,12_ = 73.798, *P < *0.001; CA4: *F*_2,12_ = 26.518, *P < *0.001) (Fig. 5D to F) or 1× (CA2: *F*_2,12_ = 157.646, *P < *0.001; CA3: *F*_2,12_ = 159.995, *P < *0.001; CA4: *F*_2,12_ = 87.663, *P < *0.001) (Fig. 5A to C) Lys diet.

**FIG 6:**
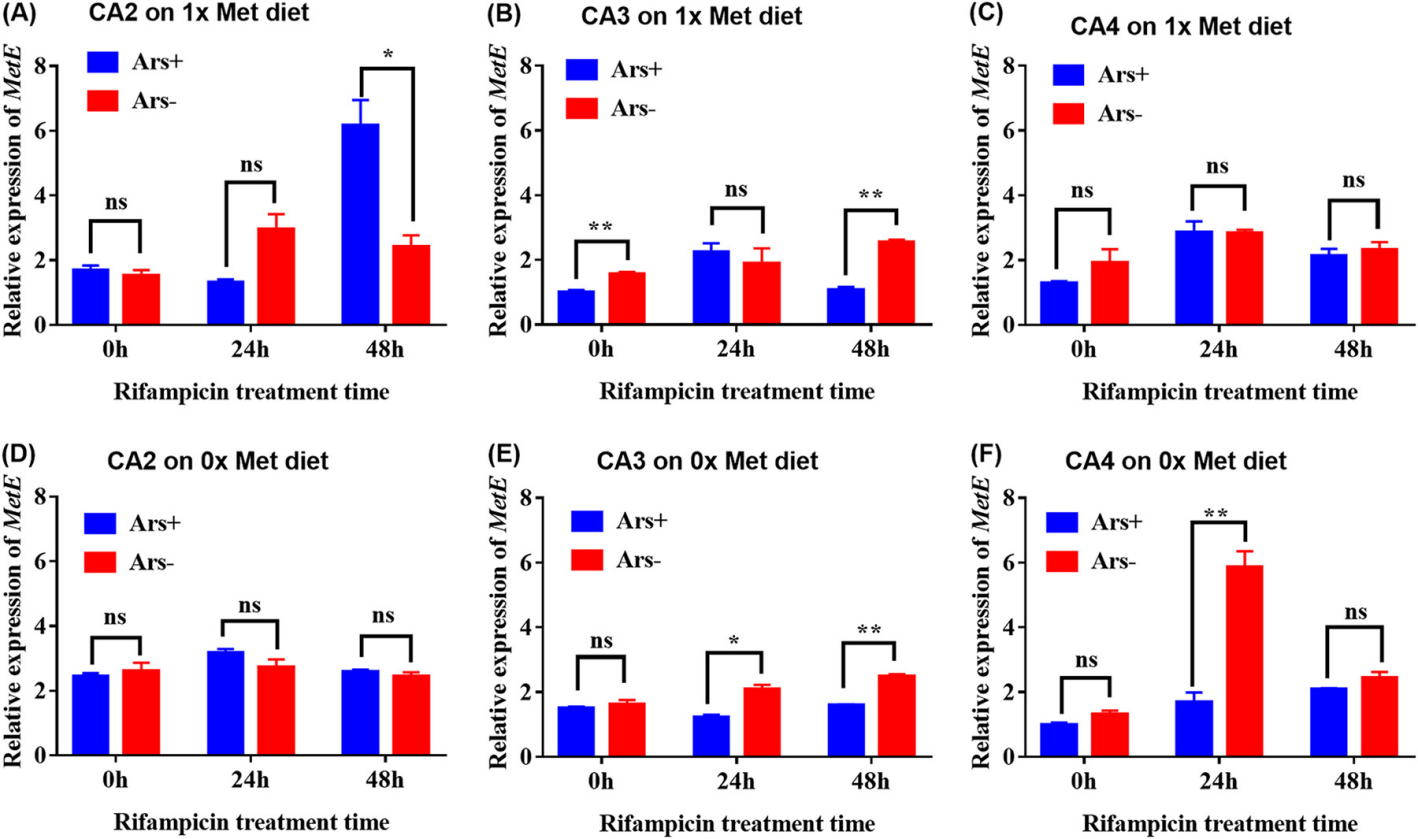
Effect of *Arsenophonus* infection on the expression level of the amino acid synthase gene *metE* of *Buchnera* in aphids reared on the 1× (A to C) and 0× (D to F) Met diets when their *Buchnera* levels were normal (no rifampicin treatment [0 h]) and reduced by using rifampicin for 24 h and 48 h. Differences in the relative expression level of the *Buchnera* gene between the Ars^+^ and Ars^−^ lines were analyzed by Student’s *t* test. *, *P* = 0.05; **, *P* = 0.01; ns, no significant difference (*P* = 0.05).

The relative expression levels of *metE* of *Buchnera*, a Met synthase gene, in the CA2 and CA4 aphids were not affected by *Arsenophonus* infections when aphids were reared on the 0× and 1× Met diets with normal *Buchnera* abundance (Fig. 6A, C, D, and F). In the CA3 line, *Arsenophonus* infections inhibited the expression level of *metE* when aphids were reared on the 1× Met diet with normal *Buchnera* abundance (*t *= 5.943, *df *= 4, *P* = 0.004) (Fig. 6B), but on the 0× Met diet, the inhibition disappeared (*t *= 0.868, *df *= 4, *P* = 0.435) (Fig. 6E). However, when *Buchnera* was partially removed by antibiotic treatment for 48 h, *Arsenophonus* infections also significantly inhibited the expression level of *metE* in the CA3 on both the 1× (*t *= 11.794, *df *= 4, *P < *0.001) (Fig. 6B) and 0× (*t *= 10.462, *df *= 4, *P < *0.001) (Fig. 6E) Met diets. The expression level of *metE* in the CA3 line was significantly affected by the interaction between *Arsenophonus* infections and *Buchnera* abundance when aphids were reared on 0× (*F*_2,12_ = 9.662, *P* = 0.003) and 1× (*F*_2,12_ = 7.642, *P* = 0.007) Met diets (Fig. 6B and E).

### Location of *Arsenophonus* and *Buchnera* in aphids.

**FIG 7:**
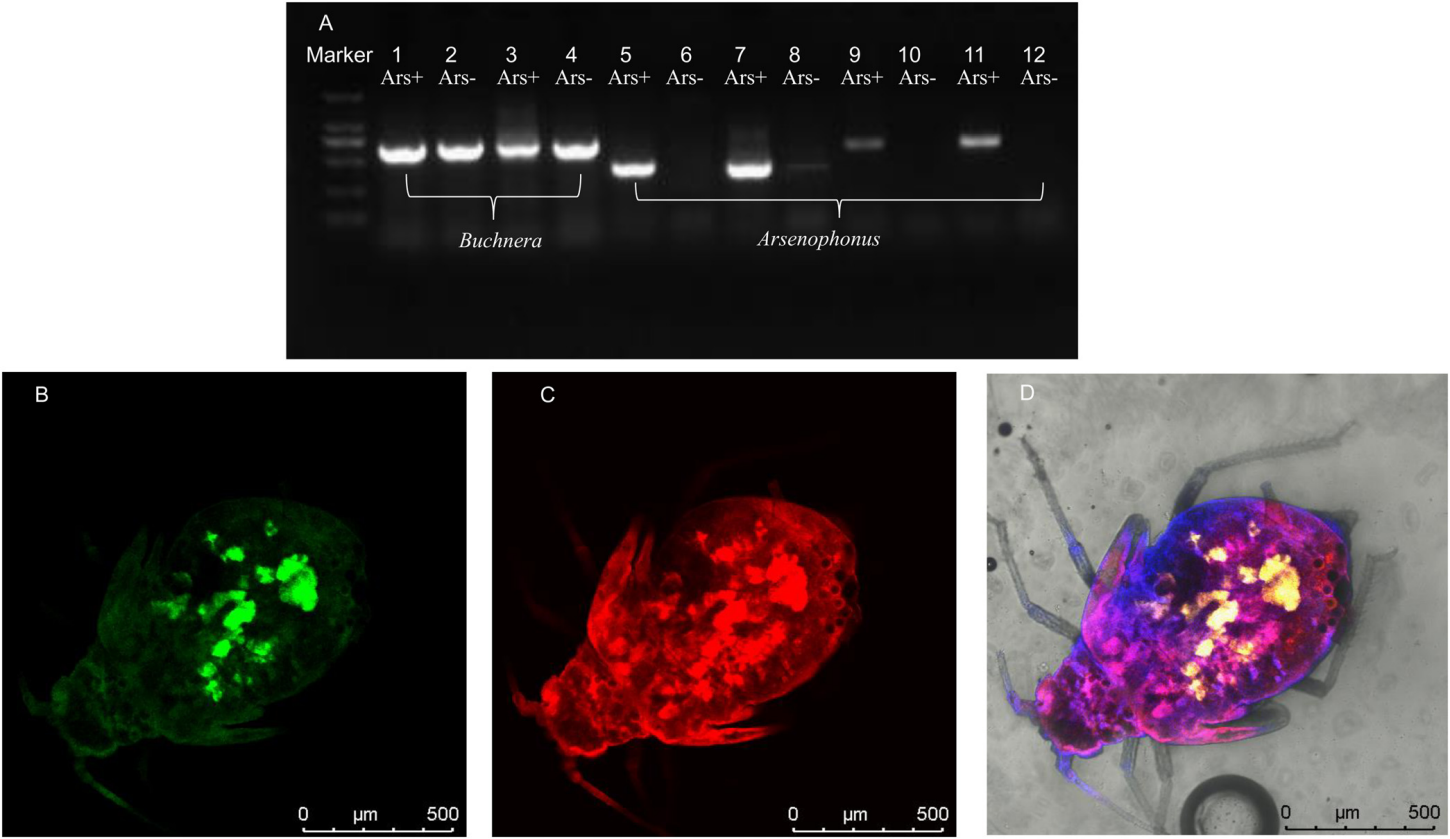
Presence of *Buchnera* and *Arsenophonus* in bacteriocytes of CA3 aphids detected using diagnostic PCR based on the Buc1 primer for *Buchnera* (lanes 1 to 4) and 16S rRNA (lanes 5 to 8) and *fbaA* (lanes 9 to 12) primers (Table S1) for *Arsenophonus* in the CA3 line (A) and detection using FISH (B to D). (B) *Buchnera*; (C) *Arsenophonus*; (D) merge of images in panels B and C. Lanes 1, 3, 5, 7, 9, and 11, *Arsenophonus*-infected aphids (Ars^+^); lanes 2, 4, 6, 8, 10, and 12, *Arsenophonus*-cured aphids (Ars^−^). For lanes 1, 2, 5, 6, 9, and 10, DNA samples were collected from an aphid body, and for lanes 3, 4, 7, 8, 11, and 12, DNA samples were collected from bacteriocytes in aphids. The primer based on *fbaA* is more specific to *Arsenophonus* than that based on 16S rRNA.

We dissected aphids to collect bacteriocytes. PCR based on specific primers for *Buchnera* and *Arsenophonus* detected both the endosymbionts not only in the bodies of the *Arsenophonus*-infected aphids but also in their bacteriocytes (Fig. 7A; Fig. S1). Fluorescence *in situ* hybridization (FISH) also showed the colocalization of *Arsenophonus* and *Buchnera* (Fig. 7B to D). Both the experimental results suggest that *Arsenophonus* coexists in bacteriocytes with *Buchnera* and that interaction between them may be possible.

## DISCUSSION

The facultative endosymbiont *Arsenophonus* exhibits a beneficial effect on aphid populations, increasing the ability of *Aphis craccivora* to use the host plant, locust (38), improving the reproduction of *Aphis glycines* (28), and enhancing the net reproductive rate and intrinsic rate of natural increase of *A. gossypii* populations (16, 30). Here, we found that *Arsenophonus* infections improved growth performance of *A. gossypii* under amino acid deficiency stress. Among aphids growing on the Lys- or Met-deficient diet, the *Arsenophonus*-infected lines were generally heavier than their *Arsenophonus*-cured counterparts, but on the normal and excess amino acid diets, their body weights were similar, suggesting that *Arsenophonus* is responsible for aphid growth under amino acid stress. Amino acid in the diet is related to aphid growth. Omission of methionine in the diet led to growth arrest of Myzus persicae, and omission of lysine caused slower growth (39). In some of the aphid genotypes used in the present study, the titers of Arg, Phe, and Trp were higher in the Ars^+^ line than in the Ars^−^ line, but titers of Lys and Met were lower. The contents of amino acids in cotton and cucumber leaves are different, and those contents are usually lower in cotton than that in cucumber (14). In contrast, infection rates of *Arsenophonus* in aphids on cotton are higher than those in aphids on cucumber, and moreover, *Arsenophonus* infections alter the requirements of aphids for specific amino acids (12). Therefore, we suggested that the growth regulation of nymphal aphids by *Arsenophonus* might be associated with the provision of amino acids.

The obligate symbiont *Buchnera* synthesizes amino acids in aphids (40). Those amino acids—Arg, Lys, Phe, Leu, and Met—are almost absent in cotton leaves (14), but all of them were found in the Ars^+^ and Ars^−^ aphid lines with a relatively high titer when aphids were fed on cotton leaves. The infection with *Arsenophonus* did not result in the same effect on the amino acid titer in aphids of different genotypes, suggesting that *Arsenophonus* does not directly supply amino acids to aphids (41, 42). The amino acid stress-dependent growth amelioration of *Arsenophonus*-infected aphids may be associated with the obligate symbiont *Buchnera*.

The abundance of an endosymbiont may affect its role in host insects (43–45). The relative abundance of *Buchnera* is affected by host aphids, environmental factors, and interaction with other symbionts in hosts (31, 33, 46, 47). Aphids feeding on cucumber plants harbored more *Buchnera* than those on cotton (33). High temperature inhibited the development of the *Buchnera* population in aphids (46, 48). Coinfections with facultative symbionts increased or decreased the population density of *Buchnera* depending on the species of facultative symbiont (21, 31, 49). In this study, the relative abundance of *Buchnera* was strongly suppressed by *Arsenophonus* when aphids were reared on the normal amino acid diet, but on the Lys- or Met-deficient diet, the presence of *Arsenophonus* did not affect the relative abundance of *Buchnera*. Also, on the original host plant, cotton, *Arsenophonus* infections did not affect the relative abundance of *Buchnera* (14). A previous study showed that the fecundity of aphids on the normal amino acid diet used in this study was lower than that on host plant leaves (30). These results show that the normal amino acid diet used in this study is not optimal for the CA2, CA3, and CA4 aphids. The conditioned effect of facultative symbionts on obligate symbionts was also found in the pea aphid, and the facultative symbiont *Regiella* or “*Candidatus* Fukatsuia” enhanced the recovery of population density of *Buchnera* after heat shock (48). The effect of *Arsenophonus* on the abundance of the amino acid synthesis-related symbiont *Buchnera* may be a pathway for aphids to deal with amino acid stress in their diet, and consequently, *Arsenophonus* infections alter the requirements of aphids for some specific amino acids (14) and improve the growth performance of aphids.

However, the effect of *Arsenophonus* on aphid growth performance was not determined by the relative abundance of endosymbionts. Although *Buchnera* was positively correlated with *Arsenophonus* in terms of relative abundance, their abundance was not correlated with the body weight of aphids. A previous study showed that the removal of *Arsenophonus* delayed the development of immature stages and reduced nymphal survival rates of the date palm hopper, Ommatissus lybicus (50). Elimination of *Arsenophonus* and “*Ca.* Hamiltonella” shortened the total life span of *A. gossypii* (16). The presence of *Arsenophonus* is beneficial to aphids’ development and survival. *Buchnera* is distributed in specific bacteriocytes in aphids (51). *Arsenophonus* was also restricted to the bacteriocytes, which the obligatory symbiont “*Candidatus* Portiera” occupies in whiteflies (2, 52, 53). Here, we found the coexistence of *Arsenophonus* and *Buchnera* in bacteriocytes in *A. gossypii*. The coexistence and correlation in population dynamics imply that there may be interaction between *Arsenophonus* and *Buchnera*.

The interaction between *Arsenophonus* and *Buchnera* affected the expression level of the amino acid synthase genes of *Buchnera*. *Arsenophonus* infection may affect amino acid synthesis in *Buchnera* to enhance the adaptability of aphids to amino acid stress in the diet and thus improve growth performance of aphids. The *Buchnera* genome contains most genes for biosynthesis of essential amino acids, such as Arg, Lys, Met, and Trp (6, 40, 54, 55). *Arsenophonus* infections affected the relative expression levels of the Lys and Met synthase genes *lysA* and *metE* of *Buchnera*, although this effect was still dependent on aphid genotype. Moreover, these *Arsenophonus*-mediated changes in gene expression levels were different in aphids reared on the normal and deficient amino acid diets, suggesting that *Arsenophonus* is likely to play a role for aphids in dealing with amino acid stress. In CA2 aphids, the *lysA* expression level was significantly higher in the *Arsenophonus*-infected line than that in its *Arsenophonus*-cured line when aphids were reared on the Lys-deficient diet. The *metE* expression level was significantly lower in the *Arsenophonus*-infected CA3 line than in the *Arsenophonus*-cured line on the normal amino acid diet, but it became equal on the Met-deficient diet. Moreover, when *Buchnera* was suppressed by an antibiotic, the effect of *Arsenophonus* on the gene expression level of amino acid synthases of *Buchnera* also changed remarkably. These results suggest that *Arsenophonus* may prompt the change of the amino acid synthesis level of *Buchnera* when aphids undergo amino acid stress.

The *Buchnera* genome completely lost various regulatory systems, such as regulatory genes of biosynthesis of essential amino acids, indicating that *Buchnera* does not respond to environmental changes (55, 56). Therefore, the expression regulation of amino acid synthase genes of *Buchnera* may likely be controlled by hosts or other coinfecting symbionts. The aphid genotype-dependent effects of *Arsenophonus* on abundance and gene expression level of *Buchnera* imply that the regulation of amino acid synthase genes may be mediated by host aphids. The *Arsenophonus* genome does not carry a whole set of amino acid synthase genes (41, 42), meaning that this symbiont is unable to supply amino acids for aphids, but it affected the expression level of the amino acid synthase genes of *Buchnera* and altered aphid performance under amino acid deficiency stress. Therefore, *Arsenophonus* affected the regulation of *Buchnera* genes. It is still worth studying whether this effect is a direct one, from *Arsenophonus* to *Buchnera*, or an indirect one, from *Arsenophonus* to the host aphid and then *Buchnera*.

**FIG 8:**
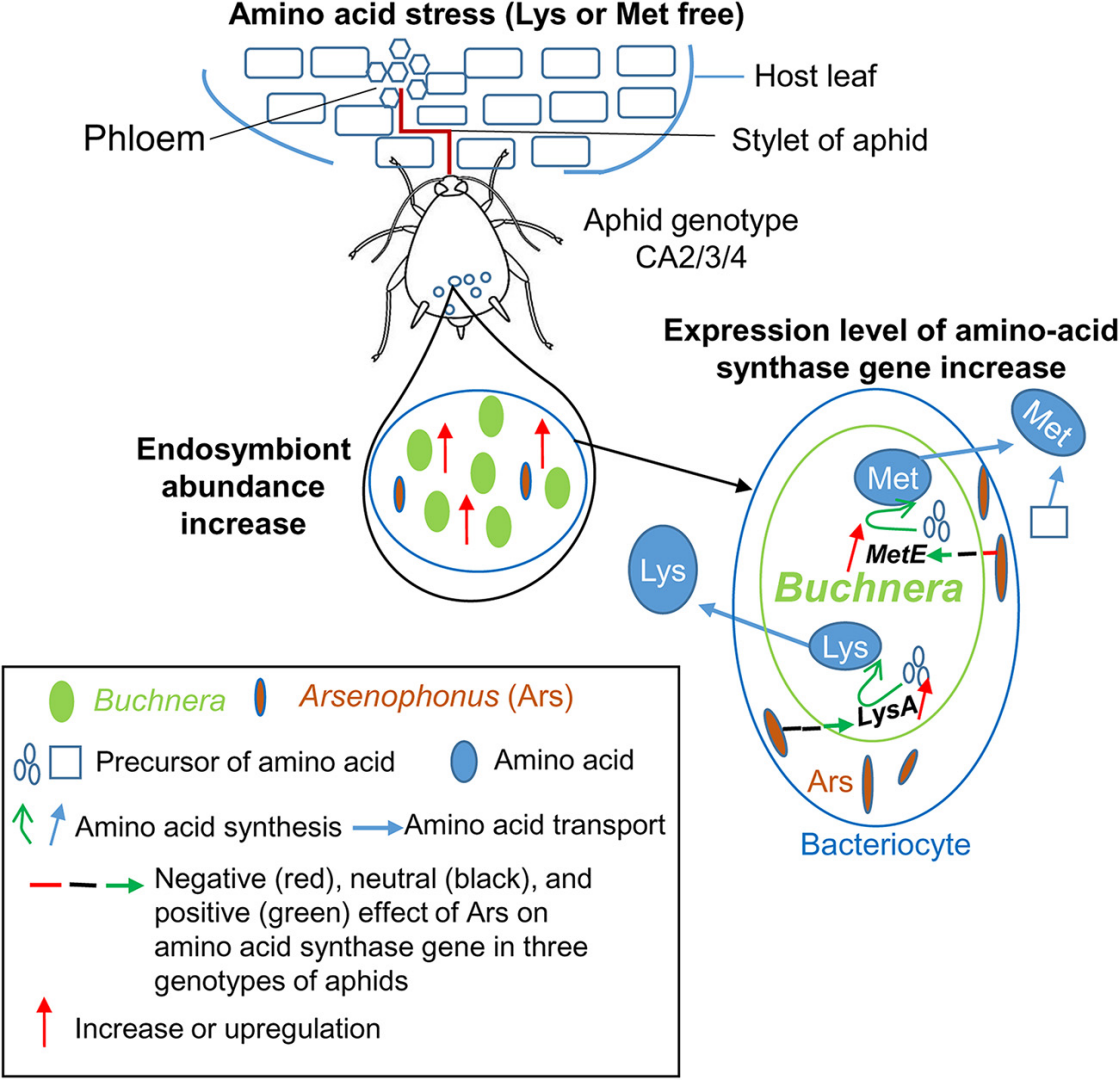
Model of the interaction between the facultative endosymbiont *Arsenophonus* and the obligate symbiont *Buchnera* to enhance aphids’ ability to adapt to amino acid stress.

It has been confirmed that *Arsenophonus*-mediated growth performance of aphids under amino acid stress concerned the abundance and gene expression level of *Buchnera*. When aphids undergo amino acid stress, *Arsenophonus* abundance changes, and this is linked to changes in *Buchnera*. On the other hand, *Arsenophonus* infections lead to the change in expression levels of amino acid synthase genes of *Buchnera* which directly increase or decrease the supply of amino acids for aphids. *Arsenophonus* improves aphid growth performance under amino acid stress, possibly via interaction with the obligate symbiont *Buchnera* (Fig. 8). The higher abundance of *Buchnera* and high expression levels of amino acid synthase genes may result in the higher synthesis of amino acids which aphids require. The finding that the effect of *Arsenophonus* on expression levels of amino acid synthase genes in the aphids with reduced levels of *Buchnera* was not the same as that in the aphids with normal levels of *Buchnera* indicates that the role of *Arsenophonus* is indeed dependent on the abundance of *Buchnera*.

Effects of *Arsenophonus* on growth performance of aphids and the relative abundance and expression levels of amino acid synthase genes of *Buchnera* are dependent on aphid genotype. The aphid genotype-dependent roles of *Arsenophonus* may be attributable to the strain of symbiont or to the aphids’ requirements for amino acids. It has been found that the S-type *Arsenophonus* strain significantly decreased the insecticide resistance of the brown planthopper Nilaparvata lugens, but the N-type strain did not (57). In this study, the *Arsenophonus* strains in the three genotypes of aphids had slightly different *fbaA* and 16S rRNA gene sequences (30). This small difference may change the role of *Arsenophonus* in hosts, and it is worth exploring. Notably, we found that the titers of amino acid in three genotypes on cotton leaves were not the same, suggesting that requirements of aphids for amino acids are different. A symbiont which can meet the requirement of aphids in a specific environment may be reserved under natural selection. Due to various requirements of aphids, the significance of *Arsenophonus*-mediated improvement of growth performance was not wholly similar among different genotypes under a specific amino acid stress. Therefore, *Arsenophonus*-mediated changes in the population abundance and amino acid synthase gene expression of *Buchnera* were also dependent on aphid genotype.

## MATERIALS AND METHODS

### Cotton-melon aphids and host plants.

Three genotypes of the cotton-melon aphid, Aphis gossypii (CA2, CA3, and CA4, based on the polymorphisms of six microsatellite loci), which had been collected from cotton and reared in the laboratory 4 years ago were used in this study (14). Aphids of the three genotypes were infected with *Arsenophonus*, one of nine known facultative symbionts; this was tested frequently using the diagnostic PCR method (14), which confirmed that *Arsenophonus* was still present. Therefore, these three genotypes are called the *Arsenophonus*-infected (Ars^+^) lines. The *fbaA* and 16S rRNA gene sequences of *Arsenophonus* in these three genotypes had a high degree of similarity, and only one and four mutations of bases, respectively, were found (30). We added antibiotics (400 μg/mL ampicillin, 200 μg/mL cephalosporin, and 200 μg/mL gentamicin) to the artificial diet to cure the *Arsenophonus* infection of the three Ars^+^ lines 3 years ago and tested them frequently to confirm that the *Arsenophonus* was still absent recently; thus, the three corresponding *Arsenophonus*-cured (Ars^−^) lines were established. The antibiotics used to cure the *Arsenophonus* infections in the CA2, CA3, and CA4 genotypes did not affect the abundance of *Buchnera* when aphids were reared on cotton leaves (14). All Ars^+^ and Ars^−^ aphid lines were reared on cotton leaves at 25°C with a photoperiod of 14 h of light and 10 h of darkness.

### Examination of amino acids in aphids.

One hundred 6-day-old aphids each of the CA2, CA3, and CA4 genotypes in the Ars^+^ and Ars^−^ lines reared on cotton leaves were collected, weighed, and put into an Eppendorf micro test (EP) tube. One milliliter of 0.02 N HCl was added, and the aphids were ground. The aphid mixture was kept at 4°C for 6 h and then centrifuged at 14,000 rpm at 4°C for 15 min. A 500-μL portion of supernatant fluid was transferred into a tube and centrifuged at 14,000 rpm at 4°C for 15 min. The supernatant fluid was filtered using a 0.22-μm membrane and used to examine the titers of amino acids. Seventeen amino acids (Fig. 1) were measured using a Hitachi automatic amino acid analyzer (L-8900; Hitachi, Japan) by the method of Tian et al. (14). Three replicates were performed for each genotype in each line.

### Measurement of body weight of aphids.

To control amino acid intake, an artificial diet (14) was used to rear aphids. Here, two amino acids, lysine (Lys) and methionine (Met), in the diet were controlled, and each amino acid was used at three concentrations, 0, 1, and 5 times the amount in the referenced diet (14). The Ars^+^ and Ars^−^ aphid lines of the CA2, CA3, and CA4 genotypes were reared using these amino acid-controlled diets to explore the effect of *Arsenophonus* infections on aphid growth under amino acid stress. Ten newborn aphids were reared on each kind of amino acid-controlled artificial diet using two layers of thin Parafilm (58), and 40 replicates were performed for each aphid line on each kind of artificial diet. One hundred 3-day-old aphids reared on one kind of amino acid diet were collected as a sample to measure their total body weight using a Sartorius BSA 124S-CW (0.1 mg) balance to exhibit the growth performance of aphids. Three or four samples were collected and measured for each aphid line on each amino acid-controlled diet. These 3-day-old aphids were also collected and stored at −80°C for the examination of the relative abundance of *Buchnera* and relative expression levels of genes using real-time qPCR.

### Examination of the relative abundances of *Arsenophonus* and *Buchnera* in aphids.

The relative abundance of *Arsenophonus* was examined following the method of Chang et al. (30). Copy numbers of the *Arsenophonus ftsK* gene and the aphid *ef1α* gene were detected using qPCR. The ratio of *ftsK* copy number to that of *ef1α* was considered the relative abundance of *Arsenophonus* in aphids. Similarly, the absolute copy numbers of the *groEL* gene of *Buchnera* and the *ef1α* gene of aphids were also examined, and the relative abundance of *Buchnera* in aphids was estimated using the ratio of the *groEL* copy number to that of *ef1α* (14). Five *Arsenophonus*-infected and *Arsenophonus*-cured aphids of the CA2, CA3, and CA4 genotypes reared using controlled amino acid diets with 0×, 1×, and 5× amino acids for 3 days were collected as samples during the body weight experiment described above, and their DNA was extracted to examine the relative abundance of *Arsenophonus* and *Buchnera*. Three separate biological repeats were performed for the Ars^+^ and Ars^−^ lines of the genotypes CA2, CA3, and CA4 reared on two kinds of amino acid-controlled diet (Lys and Met). qPCR followed the method of Tian et al. (14) using SYBR premix Ex Taq (TaKaRa) in an ABI 7500 real-time PCR system (Thermo Fisher Scientific) using the *ftsK* forward primer *ftsK*-F (5′-TCAAGGTGGCGCTGAATCTT-3′) and the reverse primer *ftsK*-R (5′-CGGGCTTACCTCTAGCTTTCC-3′), with 103% amplification efficiency; the *groEL* primers *GroEL*-F (5′-GCCATCCAAAGCCGTATTAGT CA-3′) and *GroEL*-R (5′-AGTACCGCAACACCACCAGATA-3′), with 107% amplification efficiency; and the *ef1α* primers *ef1α*-F (5′-TCACCATCATTGACG CACCTG-3′) and *ef1α*-R (5′-CCAGTACCAGCAGCAACGAT AAG-3′), with 108% amplification efficiency (14). The qPCR procedures were 95°C for 0.5 min, followed by 40 cycles of 95°C for 5 s and 60°C for 34 s. Each DNA sample was run in three separate qPCRs as technical repeats. Standard curves (log concentration of DNA on the *x* axis and PCR cycle number on the *y* axis) for the *ftsK*, *groEL*, and *ef1α* genes were set up based on the method of Zhang et al. (33). The absolute copy numbers of *groEL* and *ef1α* in each sample were computed by the method of Whelan et al. (59).

### Detection of the expression levels of amino acid synthase genes of *Buchnera*.

Five aphids reared on one amino acid-controlled diet for 3 days were pooled in one RNase-free EP tube, and there were 3 biological repeats of each. Three hundred microliters of TRIzol and zirconia beads was put into the tube and used to grind the aphids. Total RNA of the five aphids was extracted using the TRIzol reagent (TaKaRa, Japan). The integrity of extracted RNA was tested by agarose gel electrophoresis, and its concentration met the standard for qPCR. Then, cDNA synthesis was carried out according to TaKaRa PrimeScript RT reagent kit with gDNA Eraser (TaKaRa, Japan). The synthesized cDNA was stored at −80°C for qPCR to detect the relative expression levels of amino acid synthase genes of *Buchnera*.

Two genes of *Buchnera* were chosen, *lysA* and *metE*, which encode the amino acid synthesis of Lys and Met, respectively (40). Their relative expression levels in the Ars^+^ and Ars^−^ aphids on the amino-acid-controlled diet were examined using a qPCR method based on two reference genes, the actin gene and *ef1α*, of aphids (48, 60, 61). The qPCR was performed in a thermocycler (no. 7500; Applied Biosystems, Carlsbad, CA) according to the instructions for the SYBR premix Ex Taq (TaKaRa). The PCR volume was 20 μL, containing 10 μL SYBR green PCR master mix (TaKaRa), 2 μL cDNA templates, 0.4 μL forward and reverse primers (10 μM), 0.4 μL ROX reference dye II, and 6.8 μL double-distilled H_2_O (Table S1). The qPCR programs were 95°C for 30 s, followed by 40 cycles of 95°C for 5 s and 60°C for 34 s. Each qPCR was performed in three technical replicates. The relative expression level of a gene was calculated using the 2^−ΔΔ^*^CT^* method (62).

To explore the interaction between *Arsenophonus* and *Buchnera*, the Ars^+^ and Ars^−^ lines were reared on an artificial diet with 2 μg/mL rifampicin for 24 h and 48 h to reduce the abundance of *Buchnera*, and then these aphids were reared on 0× and 1× Lys and Met diets for 3 days. The aphid samples were collected to examine the relative expression levels of *lysA* and *metE* using qPCR as described above. Three biological repeats were performed for rifampicin and amino acid treatments for CA2, CA3, and CA4.

### Location of *Arsenophonus* and *Buchnera*.

We used diagnostic PCR and FISH to examine the location of *Arsenophonus* and *Buchnera* in aphids. DNA samples from an aphid body and 1,000 bacteriocytes collected from aphids according to the method of Douglas and Dixon (63) were extracted. Bacteriocytes were washed three times using absolute alcohol before DNA extraction. DNA from the *Arsenophonus*-infected and cured CA2, CA3, and CA4 aphids was extracted. The PCR detection of *Buchnera* used the primers Buc1F and Buc1R, and the detection of *Arsenophonus* used two pairs of primers based on 16S rRNA and *fbaA* (Table S1). PCR products were examined using agarose gel electrophoresis to confirm the presence or absence of *Buchnera* and *Arsenophonus* in the DNA samples. If both *Buchnera* and *Arsenophonus* were detected in bacteriocytes, it indicated that these two endosymbionts coexist in the same bacteriocytes. *Buchnera* and *Arsenophonus* were visualized in CA2, CA3, and CA4 aphids using FISH as described previously (64). The fluorochrome-labeled oligonucleotide probes for *Buchnera* and *Arsenophonus* were 5′-Cy3-CCTCTTTTGGGTAGATCC-3′ and 5′-Cy5-CCTTAACACCTTCCTCACGAC-3′, respectively (21, 65, 66).

### Data analyses.

Effects of aphid genotype and *Arsenophonus* infection on amino acid titers in aphid bodies were analyzed using multivariate analysis of variance (MANOVA) in the general linear model (GLM), and the titers of 17 amino acids were dependent variables. The difference between amino acid titers in the Ars^+^ and Ars^−^ lines was analyzed using Student’s *t* test. The effect of *Arsenophonus* infection on body weights of aphids of a given genotype was analyzed using Student’s *t* test followed by the Bonferroni correction between the Ars^+^ and Ars^−^ lines reared on the diets containing 0×, 1×, and 5× concentrations of one amino acid. The effects of amino acid deficiency or excess and aphid genotype on the relative abundance of *Arsenophonus* in aphids were analyzed using two-way ANOVA in the GLM, and the differences in the relative abundance of *Arsenophonus* in an aphid genotype reared on different amino acid-controlled diets were analyzed using ANOVA followed by the *post hoc* Tukey’s test. The effect of aphid genotype and *Arsenophonus* infection on the relative abundance of *Buchnera* in aphids on a 1× amino acid diet was analyzed using the GLM. The difference in the relative abundance of *Buchnera* between Ars^+^ and Ars^−^ lines was analyzed using Student’s *t* test followed by the Bonferroni correction. The correlation analyses between the relative abundance of *Arsenophonus* and *Buchnera* in all three genotypes (CA2, CA3, and CA4) on diets with deficient (0×), normal (1×), and excess (5×) Lys and Met and between the relative abundance of *Arsenophonus* or *Buchnera* and aphid body weight were performed using the Pearson method, and the Ars^+^ and Ars^−^ lines were analyzed separately when data collected from aphids reared on 0×, 1×, and 5× amino acid diets were pooled. Effects of *Arsenophonus* infection and *Buchnera* treatment time by antibiotic on the relative expression levels of amino acid synthase genes of *Buchnera* in aphids were analyzed using the GLM, and the difference between Ars^+^ and Ars^−^ lines was checked by Student’s *t* test. All data analyses were performed using IBM SPSS Statistics V25.

## References

[1] Tsuchida T, Koga R, Shibao H, Matsumoto T, Fukatsu T. 2002. Diversity and geographic distribution of secondary endosymbiotic bacteria in natural populations of the pea aphid, Acyrthosiphon pisum. Mol Ecol 11:2123–2135. doi:10.1046/j.1365-294x.2002.01606.x. 12296954

[2] Skaljac M, Zanic K, Ban SG, Kontsedalov S, Ghanim M. 2010. Co-infection and localization of secondary symbionts in two whitefly species. BMC Microbiol 10:142. doi:10.1186/1471-2180-10-142. 20462452 PMC2877686

[3] Toju H, Fukatsu T. 2011. Diversity and infection prevalence of endosymbionts in natural populations of the chestnut weevile: relevance of local climate and host plants. Mol Ecol 20:853–868. doi:10.1111/j.1365-294X.2010.04980.x. 21199036

[4] Zhou JC, Zhao X, Huo LX, Shang D, Dong H, Zhang LS. 2022. Wolbachia-driven memory loss in a parasitic wasp increases superparasitism to enhance horizontal transmission. mBio 13:e02362-22. doi:10.1128/mbio.02362-22. 36214563 PMC9765423

[5] Kang ZW, Zhang M, Cao HH, Guo SS, Liu FH, Liu TX. 2022. Facultative endosymbiont Serratia symbiotica inhibits the apterization of pea aphid to enhance its spread. Microbiol Spectr 10:e04066-22. doi:10.1128/spectrum.04066-22. 36445124 PMC9769995

[6] Douglas AE, Prosser WA. 1992. Synthesis of the essential amino acid tryptophan in the pea aphid (Acyrthosiphon pisum) symbiosis. J Insect Physiol 38:565–568. doi:10.1016/0022-1910(92)90107-O.

[7] Sandström J, Pettersson J. 1994. Amino acid composition of phloem sap and the relation to intraspecific variation in pea aphid (Acyrthosiphon pisum) performance. J Insect Physiol 40:947–955. doi:10.1016/0022-1910(94)90133-3.

[8] Karley A, Douglas A, Parker W. 2002. Amino acid composition and nutritional quality of potato leaf phloem sap for aphids. J Exp Biol 205:3009–3018. doi:10.1242/jeb.205.19.3009. 12200404

[9] Moran NA, Munson MA, Baumann P, Ishikawa H. 1993. A molecular clock in endosymbiotic bacteria is calibrated using the insect hosts. Proc R Soc B 253:167–171.

[10] Montllor C, Maxmen A, Purcell AH. 2002. Facultative bacterial endosymbionts benefit pea aphids Acyrthosiphon pisum under heat stress. Ecol Entomol 27:189–195. doi:10.1046/j.1365-2311.2002.00393.x.

[11] Łukasik P, van Asch M, Guo H, Ferrari J, Godfray HC. 2013. Unrelated facultative endosymbionts protect aphids against a fungal pathogen. Ecol Lett 16:214–218. doi:10.1111/ele.12031. 23137173

[12] Doremus MR, Oliver KM. 2017. Aphid heritable symbiont exploits defensive mutualism. Appl Environ Microbiol 83:e03276-16. doi:10.1128/AEM.03276-16. 28159793 PMC5377491

[13] Wang Q, Yuan E, Ling X, Zhu-Salzman K, Guo H, Ge F, Sun Y. 2020. An aphid facultative symbiont suppresses plant defence by manipulating aphid gene expression in salivary glands. Plant Cell Environ 43:2311–2322. doi:10.1111/pce.13836. 32596816

[14] Tian PP, Chang CY, Miao NH, Li MY, Liu XD. 2019. Infections with Arsenophonus facultative endosymbionts alter performance of aphids (Aphis gossypii) on an amino-acid-deficient diet. Appl Environ Microbiol 85:e01407-19. doi:10.1128/AEM.01407-19. 31540983 PMC6856319

[15] Gomez-Valero L, Soriano-Navarro M, Perez-Brocal V, Heddi A, Moya A, Garcia-Verdugo JM, Latorre A. 2004. Coexistence of Wolbachia with Buchnera aphidicola and a secondary symbiont in the aphid Cinara cedri. J Bacteriol 186:6626–6633. doi:10.1128/JB.186.19.6626-6633.2004. 15375144 PMC516615

[16] Ayoubi A, Talebi AA, Fathipour Y, Mehrabadi M. 2020. Coinfection of the secondary symbionts, Hamiltonella defensa and Arsenophonus sp. contribute to the performance of the major aphid pest, Aphis gossypii (Hemiptera: Aphididae). Insect Sci 27:86–98. doi:10.1111/1744-7917.12603. 29749703

[17] McLean AHC, Parker BJ, Hrcek J, Kavanagh JC, Wellham PAD, Godfray HCJ. 2018. Consequences of symbiont co-infections for insect host phenotypes. J Anim Ecol 87:478–488. doi:10.1111/1365-2656.12705. 28542979

[18] Liu XD, Lei HX, Chen FF. 2019. Infection pattern and negative effects of a facultative endosymbiont on its insect host are environment-dependent. Sci Rep 9:4013. doi:10.1038/s41598-019-40607-5. 30850675 PMC6408509

[19] Weldon SR, Russell JA, Oliver KM. 2020. More is not always better: coinfections with defensive symbionts generate highly variable outcomes. Appl Environ Microbiol 86:e02537-19. doi:10.1128/AEM.02537-19. 31862723 PMC7028961

[20] Smee MR, Raines SA, Ferrari J. 2021. Genetic identity and genotype×genotype interactions between symbionts outweigh species level effects in an insect microbiome. ISME J 15:2537–2546. doi:10.1038/s41396-021-00943-9. 33712703 PMC8397793

[21] Koga R, Tsuchida T, Fukatsu T. 2003. Changing partners in an obligate symbiosis: a facultative endosymbiont can compensate for loss of the essential endosymbiont Buchnera in an aphid. Proc Biol Sci B 270:2543–2550. doi:10.1098/rspb.2003.2537. PMC1691542 14728775

[22] Lamelas A, Gosalbes MJ, Manzano-Marin A, Pereto J, Moya A, Latorre A. 2011. Serratia symbiotica from the aphid Cinara cedri: a missing link from facultative to obligate insect endosymbiont. PLoS Genet 7:e1002357. doi:10.1371/journal.pgen.1002357. 22102823 PMC3213167

[23] Li Q, Fan J, Sun J, Wang M, Chen J. 2018. Effect of the secondary symbiont Hamiltonella defensa on fitness and relative abundance of Buchnera aphidicola of wheat aphid, Sitobion miscanthi. Front Microbiol 9:582. doi:10.3389/fmicb.2018.00582. 29651279 PMC5884939

[24] Jones RT, Bressan A, Greenwell AM, Fierer N. 2011. Bacterial communities of two parthenogenetic aphid species cocolonizing two host plants across the Hawaiian Islands. Appl Environ Microbiol 77:8345–8349. doi:10.1128/AEM.05974-11. 21965398 PMC3233044

[25] Jousselin E, Cœur d’Acier A, Vanlerberghe-Masutti F, Duron O. 2013. Evolution and diversity of Arsenophonus endosymbionts in aphids. Mol Ecol 22:260–270. doi:10.1111/mec.12092. 23106652

[26] Zhao Y, Zhang S, Luo JY, Wang CY, Lv LM, Cui JJ. 2016. Bacterial communities of the cotton aphid Aphis gossypii associated with Bt cotton in northern China. Sci Rep 6:22958. doi:10.1038/srep22958. 27079679 PMC4832190

[27] Xu S, Jiang L, Qiao G, Chen J. 2020. The bacterial flora associated with the polyphagous aphid Aphis gossypii Glover (Hemiptera: Aphididae) is strongly affected by host plants. Microb Ecol 79:971–984. doi:10.1007/s00248-019-01435-2. 31802184 PMC7198476

[28] Wulff JA, White JA. 2015. The endosymbiont Arsenophonus provides a general benefit to soybean aphid (Hemiptera: Aphididae) regardless of host plant resistance (Rag). Environ Entomol 44:574–581. doi:10.1093/ee/nvv031. 26313962

[29] Brady CM, White JA. 2013. Cowpea aphid (Aphis craccivora) associated with different host plants has different facultative endosymbionts. Ecol Entomol 38:433–437. doi:10.1111/een.12020.

[30] Chang CY, Sun XW, Tian PP, Miao NH, Zhang YL, Liu XD. 2022. Plant secondary metabolite and temperature determine the prevalence of Arsenophonus endosymbionts in aphid populations. Environ Microbiol 24:3764–3776. doi:10.1111/1462-2920.15929. 35129273

[31] Sakurai M, Koga R, Tsuchida T, Meng XY, Fukatsu T. 2005. Rickettsia symbiont in the pea aphid Acyrthosiphon pisum: novel cellular tropism, effect on host fitness, and interaction with the essential symbiont Buchnera. Appl Environ Microbiol 71:4069–4075. doi:10.1128/AEM.71.7.4069-4075.2005. 16000822 PMC1168972

[32] Enders LS, Miller NJ. 2016. Stress-induced changes in abundance differ among obligate and facultative endosymbionts of the soybean aphid. Ecol Evol 6:818–829. doi:10.1002/ece3.1908. 26865969 PMC4739556

[33] Zhang YC, Cao WJ, Zhong LR, Godfray HCJ, Liu XD. 2016. Host plant determines the population size of an obligate symbiont (Buchnera aphidicola) in aphids. Appl Environ Microbiol 82:2336–2346. doi:10.1128/AEM.04131-15. 26850304 PMC4959500

[34] Liu XD, Zhai BP, Zhang XX. 2003. Studies on the host biotypes and its cause of cotton aphid in Nanjing, China. Sci Agric Sin 36:54–58.

[35] Carletto J, Lombaert E, Chavigny P, Brévault T, Lapchin L, Vanlerberghe-Masutti F. 2009. Ecological specialization of the aphid Aphis gossypii Glover on cultivated host plants. Mol Ecol 18:2198–2212. doi:10.1111/j.1365-294X.2009.04190.x. 19635073

[36] Wang L, Zhang S, Luo JY, Wang CY, Lv LM, Zhu XZ, Li CH, Cui JJ. 2016. Identification of Aphis gossypii Glover (Hemiptera: Aphididae) biotypes from different host plants in north China. PLoS One 11:e0146345. doi:10.1371/journal.pone.0146345. 26735973 PMC4703217

[37] Wang L, Zhang S, Luo J, Lu L, Wang C, Cui J. 2015. Host biotypes of cotton aphid Aphis gossypii Glover and preliminary analysis of the formation mechanism in Anyang region of China. Cotton Sci 27:372–378.

[38] Wagner SM, Martinez AJ, Ruan Y-M, Kim KL, Lenhart PA, Dehnel AC, Oliver KM, White JA. 2015. Facultative endosymbionts mediate dietary breadth in a polyphagous herbivore. Funct Ecol 29:1402–1410. doi:10.1111/1365-2435.12459.

[39] Dadd RH, Krieger DL. 1968. Dietary amino acid requirements of the aphid, Myzus persicae. J Insect Physiol 14:741–764. doi:10.1016/0022-1910(68)90186-8.

[40] Hansen AK, Moran NA. 2011. Aphid genome expression reveals host-symbiont cooperation in the production of amino acids. Proc Natl Acad Sci USA 108:2849–2854. doi:10.1073/pnas.1013465108. 21282658 PMC3041126

[41] Darby AC, Choi JH, Wilkes T, Hughes MA, Werren JH, Hurst GDD, Colbourne JK. 2010. Characteristics of the genome of Arsenophonus nasoniae, son-killer bacterium of the wasp Nasonia. Insect Mol Biol 19:75–89. doi:10.1111/j.1365-2583.2009.00950.x. 20167019

[42] Santos-Garcia D, Juravel K, Freilich S, Zchori-Fein E, Latorre A, Moya A, Morin S, Silva FJ. 2018. To B or not to B: comparative genomics suggests Arsenophonus as a source of B vitamins in whiteflies. Front Microbiol 9:2254. doi:10.3389/fmicb.2018.02254. 30319574 PMC6167482

[43] Ghanim M, Kontsedalov S. 2009. Susceptibility to insecticides in the Q biotype of Bemisia tabaci is correlated with bacterial symbiont densities. Pest Manag Sci 65:939–942. doi:10.1002/ps.1795. 19479746

[44] Lu P, Bian G, Pan X, Xi Z. 2012. Wolbachia induces density-dependent inhibition to dengue virus in mosquito cells. PLoS Negl Trop Dis 6:e1754. doi:10.1371/journal.pntd.0001754. 22848774 PMC3404113

[45] Pan HP, Chu D, Liu BM, Xie W, Wang SL, Wu QJ, Xu BY, Zhang YJ. 2013. Relative amount of symbionts in insect hosts changes with host-plant adaptation and insecticide resistance. Environ Entomol 42:74–78. doi:10.1603/EN12114. 23339787

[46] Chen CY, Lai CY, Kuo MH. 2009. Temperature effect on the growth of Buchnera endosymbiont in Aphis craccivora (Hemiptera: Aphididae). Symbiosis 49:53–59. doi:10.1007/s13199-009-0011-4.

[47] Pers D, Hansen AK. 2021. The boom and bust of the aphid’s essential amino acid metabolism across nymphal development. G3 11:jkab115. doi:10.1093/g3journal/jkab115. 33831149 PMC8433001

[48] Heyworth ER, Smee MR, Ferrari J. 2020. Aphid facultative symbionts aid recovery of their obligate symbiont and their host after heat stress. Front Ecol Evol 8:56. doi:10.3389/fevo.2020.00056.

[49] Martinez AJ, Weldon SR, Oliver KM. 2014. Effects of parasitism on aphid nutritional and protective symbioses. Mol Ecol 23:1594–1607. doi:10.1111/mec.12550. 24152321

[50] Karimi S, Seyahooei MA, Izadi H, Bagheri A, Khodaygan P. 2019. Effect of Arsenophonus endosymbiont elimination on fitness of the date palm hopper, Ommatissus lybicus (Hemiptera: Tropiduchidae). Environ Entomol 48:614–622. doi:10.1093/ee/nvz047. 31095275

[51] Nakabachi A, Shigenobu S, Sakazume N, Shiraki T, Hayashizaki Y, Carninci P, Ishikawa H, Kudo T, Fukatsu T. 2005. Transcriptome analysis of the aphid bacteriocyte, the symbiotic host cell that harbors an endocellular mutualistic bacterium, Buchnera. Proc Natl Acad Sci USA 102:5477–5482. doi:10.1073/pnas.0409034102. 15800043 PMC555734

[52] Gottlieb Y, Ghanim M, Gueguen G, Kontsedalov S, Vavre F, Fleury F, Zchori-Fein E. 2008. Inherited intracellular ecosystem: symbiotic bacteria share bacteriocytes in whiteflies. FASEB J 22:2591–2599. doi:10.1096/fj.07-101162. 18285399

[53] Skaljac M, Zanic K, Hrncic S, Radonjic S, Perovic T, Ghanim M. 2013. Diversity and localization of bacterial symbionts in three whitefly species (Hemiptera: Aleyrodidae) from the east coast of the Adriatic Sea. Bull Entomol Res 103:48–59. doi:10.1017/S0007485312000399. 22698088

[54] Sandstrom J, Moran NA. 1999. How nutritionally imbalanced is phloem sap for aphids? Entomol Exp Appl 91:203–210. doi:10.1046/j.1570-7458.1999.00485.x.

[55] Shigenobu S, Watanabe H, Hattori M, Sakaki Y, Ishikawa H. 2000. Genome sequence of the endocellular bacterial symbiont of aphids Buchnera sp. APS. Nature 407:81–86. doi:10.1038/35024074. 10993077

[56] Moran NA, Dunbar HE, Wilcox JL. 2005. Regulation of transcription in a reduced bacterial genome: nutrient-provisioning genes of the obligate symbiont Buchnera aphidicola. J Bacteriol 187:4229–4237. doi:10.1128/JB.187.12.4229-4237.2005. 15937185 PMC1151715

[57] Pang R, Chen M, Yue L, Xing K, Li T, Kang K, Liang ZK, Yuan LY, Zhang WQ. 2018. A distinct strain of Arsenophonus symbiont decreases insecticide resistance in its insect host. PLoS Genet 14:e1007725. doi:10.1371/journal.pgen.1007725. 30332402 PMC6205657

[58] Ma L, Li MY, Chang CY, Chen FF, Hu Y, Liu XD. 2019. The host range of Aphis gossypii is dependent on aphid genetic background and feeding experience. PeerJ 7:e7774. doi:10.7717/peerj.7774. 31579627 PMC6768058

[59] Whelan JA, Russell NB, Whelan MA. 2003. A method for the absolute quantification of cDNA using real-time PCR. J Immunol Methods 278:261–269. doi:10.1016/s0022-1759(03)00223-0. 12957413

[60] Yang C, Pan H, Liu Y, Zhou X. 2014. Selection of reference genes for expression analysis using quantitative real-time PCR in the pea aphid, Acyrthosiphon pisum (Harris) (Hemiptera, Aphidiae). PLoS One 9:e110454. doi:10.1371/journal.pone.0110454. 25423476 PMC4244036

[61] Zhang YC, Lei HX, Miao NH, Liu XD. 2017. Comparative transcriptional analysis of the host-specialized aphids Aphis gossypii (Hemiptera: Aphididae). J Econ Entomol 110:702–710. doi:10.1093/jee/tox029. 28334183

[62] Livak KJ, Schmittgen TD. 2001. Analysis of relative gene expression data using real-time quantitative PCR and the 2^−ΔΔCt^ method. Methods 25:402–408. doi:10.1006/meth.2001.1262. 11846609

[63] Douglas AE, Dixon AFG. 1987. The mycetocyte symbiosis of aphids: variation with age and morph in virginoparae of Megoura viciae and Acyrthosiphon pisum. J Insect Physiol 33:109–113. doi:10.1016/0022-1910(87)90082-5.

[64] Shalom SR, Weiss B, Lalzar M, Kaltenpoth M, Chiel E. 2022. Abundance and localization of symbiotic bacterial communities in the fly parasitoid Spalangia cameroni. Appl Environ Microbiol 88:e02549-21. doi:10.1128/aem.02549-21. 35420439 PMC9088259

[65] Bressan A, Terlizzi F, Credi R. 2012. Independent origins of vectored plant pathogenic bacteria from arthropod-associated Arsenophonus endosymbionts. Microb Ecol 63:628–638. doi:10.1007/s00248-011-9933-5. 21892672

[66] Tsuchida T, Koga R, Fujiwara A, Fukatsu T. 2014. Phenotypic effect of “Candidatus Rickettsiella viridis,” a facultative symbiont of the pea aphid (Acyrthosiphon pisum), and its interaction with a coexisting symbiont. Appl Environ Microbiol 80:525–533. doi:10.1128/AEM.03049-13. 24212575 PMC3911091

