# Supplementary material for: Arsenophonus Interacts with Buchnera to Improve Growth Performance of Aphids under Amino Acid Stress
Source: Microbiol Spectr. 2023 May 24;11(3):e01792-23. doi: 10.1128/spectrum.01792-23 (PMC10269474; doi:10.1128/spectrum.01792-23)
Supplement: Supplemental file 1 — Supplemental material. Download spectrum.01792-23-s0001.docx, DOCX file, 1.4 MB [file spectrum.01792-23-s0001.docx]

***Arsenophonus* interacts with *Buchnera* to ameliorate growth performance of aphid under amino acid stress**

Pan-Pan Tian, Yu-Lin Zhang, Jing-Ling Huang, Wang-Yan Li, Xiang-Dong Liu*

Department of Entomology, Nanjing Agricultural University, Nanjing, China

*Correspondence: [liuxd@njau.edu.cn](mailto:liuxd@njau.edu.cn)

**Table S1** Primer sequence used in PCR

| Gene type | Gene/symbiont | Primer name | Primer sequence (5’-3’) | Size |
| --- | --- | --- | --- | --- |
| Diagnostic PCR | *Buchnera* | Buc1F | GAATTCTAGGTGTAGCGGTGA | 660 bp |
|  |  | Buc1R | GCGATTCCGACTTCGTGGA |  |
|  | *Arsenophonus* | 16sf2 | CGGACGGGTGAGTAAGGTATG | 446 bp |
|  |  | 16sr2 | GAGTTAGCCGGTGCTTCTTCT |  |
|  | *Arsenophonus* | fbaAf | GCYGCYAAAGTTCRTTCTCC | 617 bp |
|  |  | fbaA2r | GGCAAATTAAATTTCTGCGCAACG |  |
| qPCR  reference gene | *ef1α* | EF1α-F | TCACCATCATTGACGCACCTG | 103 bp |
|  |  | EF1α-R | CCAGTACCAGCAGCAACGATAAG |  |
|  | *actin* | actin-F | TGACTTGACCGACTACTTGATG | 117 bp |
|  |  | actin-R | TCCAAAGCGACATAGCACAA |  |
| qPCR  target gene | *LysA* | LysA-F | AATCCCGTCAAACAATCGAGAGA | 180 bp |
|  |  | LysA-R | CCATATGCGCCTGTATCGTGAA |  |
|  | *metE* | metE-F | ACTTGGGCTGTCGATGCTTT | 215 bp |
|  |  | metE-R | ACACCTGGTCCCACTTCATT |  |

**Table S2** Effects of the aphid genotype and *Arsenophonus* infection on the titer of amino acid in aphid body analyzed by MANOVA

| Effect | Roy’s greatest root | *F* | *df* | | *P* |
| --- | --- | --- | --- | --- | --- |
|  |  |  | Numerator | Denominator |  |
| Genotype | 10407.252 | 1734.542 | 12 | 2 | 0.001 |
| *Arsenophonus* | 1221.738 | 101.812 | 12 | 1 | 0.077 |
| Genotype×*Arsenophonus* | 532.552 | 88.759 | 12 | 2 | 0.011 |

**Table S3** Effects of the interaction between aphid genotype and *Arsenophonus* infection on the titers of 17 amino acids in aphid body analyzed by MANOVA.

| Amino acid | Type III square sum | df | F | P |
| --- | --- | --- | --- | --- |
| **Asp** | **2110.984** | **2** | **4.698** | **0.031** |
| Glu | 29579.787 | 2 | 0.406 | 0.675 |
| **Gly** | **31028.558** | **2** | **7.778** | **0.007** |
| **Ala** | **661135.971** | **2** | **6.160** | **0.014** |
| Cys | 10093.462 | 2 | 2.453 | 0.128 |
| **Tyr** | **1910916.060** | **2** | **7.774** | **0.007** |
| **Orn** | **13.016** | **2** | **5.267** | **0.023** |
| Pro | 270818.922 | 2 | 2.838 | 0.098 |
| Val | 9834.604 | 2 | 0.712 | 0.510 |
| **Met** | **66020.551** | **2** | **7.396** | **0.008** |
| Ile | 7494.699 | 2 | 3.553 | 0.061 |
| Leu | 4.134 | 2 | 0.001 | 0.999 |
| **Phe** | **379187.181** | **2** | **8.992** | **0.004** |
| **Lys** | **174358.294** | **2** | **6.368** | **0.013** |
| His | 1212.265 | 2 | 0.174 | 0.842 |
| **Trp** | **186.092** | **2** | **17.350** | **0.000** |
| **Arg** | **89917.804** | **2** | **7.962** | **0.006** |


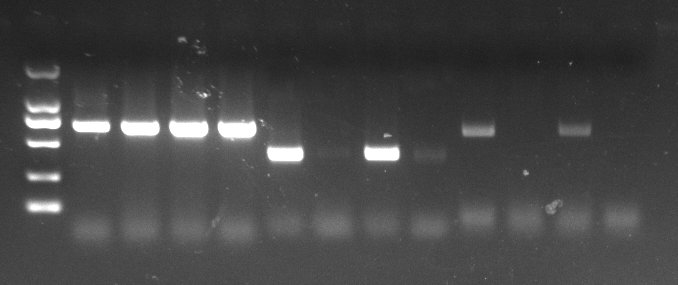


Marker 1 2 3 4 5 6 7 8 9 10 11 12

B


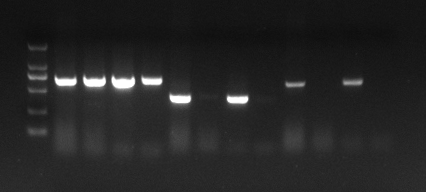


Marker 1 2 3 4 5 6 7 8 9 10 11 12

A

**Fig S1** Presence of *Buchnera* and *Arsenophonus* in bacteriocytes of CA2 (A) and CA4 (B) detected using PCR based on the Buc1 primer for *Buchnera* (line 1-4), and 16S rRNA (line 5-8) and *fbaA* (line 9-12) primers for *Arsenophonus*. Lines 1, 3, 5, 7, 9, 11 are *Arsenophonus*-infected aphids. Line 2, 4, 6, 8, 10, 12 are *Arsenophonus*-cured aphids. Lines 1, 2, 5, 6, 9, 10 DNA samples collected from an aphid body, and lines 3, 4, 7, 8, 11, 12 DNA samples collected from bacteriocytes in aphids. Both the *Buchnera* and *Arsenophonus* could be detected in the bacteriocytes and body of the *Arsenophonus*-infected aphids. The primer of *fbaA* is more specific for the *Arsenophonus* than the primer of 16S rRNA (Table S1).
